# The effect of ball mass on the mechanochemical transformation of a single-component organic system: anhydrous caffeine

**DOI:** 10.1007/s10853-018-2324-2

**Published:** 2018-04-20

**Authors:** Adam A. L. Michalchuk, Ivan A. Tumanov, Elena V. Boldyreva

**Affiliations:** 1 Novosibirsk State University, Novosibirsk, Russian Federation; 2 EaStCHEM School of Chemistry, University of Edinburgh, Edinburgh, UK; 3 EPSRC Centre for Continuous Manufacturing and Crystallisation (CMAC), Edinburgh, UK; 4 Institute of Solid State Chemistry and Mechanochemistry SB RAS, Novosibirsk, Russian Federation

## Abstract

Mechanochemical methodologies, particularly ball milling, have become commonplace in many laboratories. In the present work, we examine the effects of milling ball mass on the polymorphic conversion of anhydrous caffeine. By investigating a single-phase system, the rate-limiting step of particle–particle contact formation is eliminated. It is found that larger milling balls lead to considerably faster conversion rates. Modelling of the transformation rate suggests that a single, time-independent rate constant is insufficient to describe the transformation. Instead, a convolution of at least two rate-determining processes is required to correctly describe the transformation. This suggests that the early stages of the transformation are governed only by the number of particle–ball collisions. As the reaction proceeds, these collisions less frequently involve reactant, and the rate becomes limited by mass transport, or mixing, even in originally single-phase systems, which become multi-phase as the product is formed. Larger milling balls are less hindered by poorly mixed material. This likely results from a combination of higher impact energies and higher surface areas associated with the larger milling balls. Such insight is important for the selective and targeted design of mechanochemical processes.

**Electronic supplementary material:**

The online version of this article (10.1007/s10853-018-2324-2) contains supplementary material, which is available to authorized users.

## Introduction

Mechanochemistry has found many applications in both academic and industrial sciences. This has historically involved use in mineral processing, but is becoming increasingly common in the fine chemicals and pharmaceutical industries [1]. Mechanochemical methods have been used in the synthesis and preparation of a broad range of molecules and materials [2–5]. Many examples are now available where mechanical treatment has been shown to be a method providing access to novel molecular materials or has been used to enhance the selectivity and the rates of product formation [6–9]. In recent years, attention has largely turned towards the use of mechanical treatment as a tool for synthesis of new organic and coordination compounds, including pharmaceuticals, as well as for control of their polymorphism [10–14].

Despite the widespread interest in the use of mechanochemistry for these purposes, the mechanisms of these reactions are very poorly understood. Offline techniques are known to be affected by ‘ageing’ of mechanically treated mixtures, and ex situ methods suffer from deficiencies in sampling and disruption of powder structure (tableting). While offline techniques remain vital to mechanochemical study, their use in identifying reaction pathways and kinetics is limited. These issues prompted the rapid development of techniques to probe mechanochemical reactions in situ and in real time. To date, this has been performed using X-ray powder diffraction at synchrotron sources, laboratory-based Raman spectroscopy, or a simultaneous combination of the two [3, 15–19]. In situ, real-time monitoring has proven a great advantage to identifying processes and control parameters in mechanochemistry. In a number of examples, in situ monitoring of multi-component co-crystallisations has revealed previously unknown reaction intermediates, which in some cases could be isolated if treatment were stopped [20]. The use of in situ, real-time techniques has also allowed insights into the macroscopic rates of mechanochemical processes and has revealed a number of complex phenomena, unknown to solution and gas-phase reactions [19].

In the vast majority of cases, mechanical treatment is used to induce transformations between at least two chemically different phases. In these cases, the rate of transformation is greatly limited by the formation of heterogeneous particle–particle contacts [21]. These are required for any reaction to occur. Mixing efficiency therefore becomes one of the most important parameters of the powder sample. It follows that problems can arise in the case of rheological changes, which can have drastic effect on the flowability and mixing of the sample [19, 22]. The observed reaction rates therefore become a convolution of changes in processability with the true kinetics of the transformation. This can lead to difficulties in the identification of kinetic parameters when studying mechanochemical processes.

Despite the issues that surround kinetic analysis of multi-component transformations, many such studies have been performed. In addition to the qualitative monitoring of mechanochemical transformations, in situ technologies have been used to systematically investigate the effects of various process parameters on the rates and products of transformation. To date, this has included such effects as milling frequency, milling ball mass and temperature [17–19, 23]. This style of investigation is critical for the rational design of targeted mechanochemical procedures.

Mechanically induced polymorphism has been documented on a number of occasions, by both neat and liquid-assisted grinding. Ex situ analysis has demonstrated that the type of mechanical treatment [14, 24, 25], the addition of liquid [14, 26, 27], a polymer [28, 29] and starting polymorph [14] can all play a role in determining the outcome of a polymorphic transformation. This phenomenon is of particular importance to industry, where the solid form of a material is vital to the integrity of its function and the legalities that surround its applications. However, to the best of our knowledge, although the effect of temperature and duration of milling on a single-component transformation has been reported with reference to amorphisation [30–33] and polymorphic transformations [34–38], little work has been done to analyse the effects of other milling parameters on related processes. To the best of our knowledge, there is currently also no work dedicated to the study of single-phase polymorphic transformations under mechanical treatment, probed by real-time and in situ X-ray powder diffraction techniques. While industrially important, such investigation is of great value for fundamental mechanochemistry, where heterogeneous particle contacts are no longer required. The kinetics revealed by such a study therefore reflects the true rate of the mechanically induced conversion.

**Figure 1:**
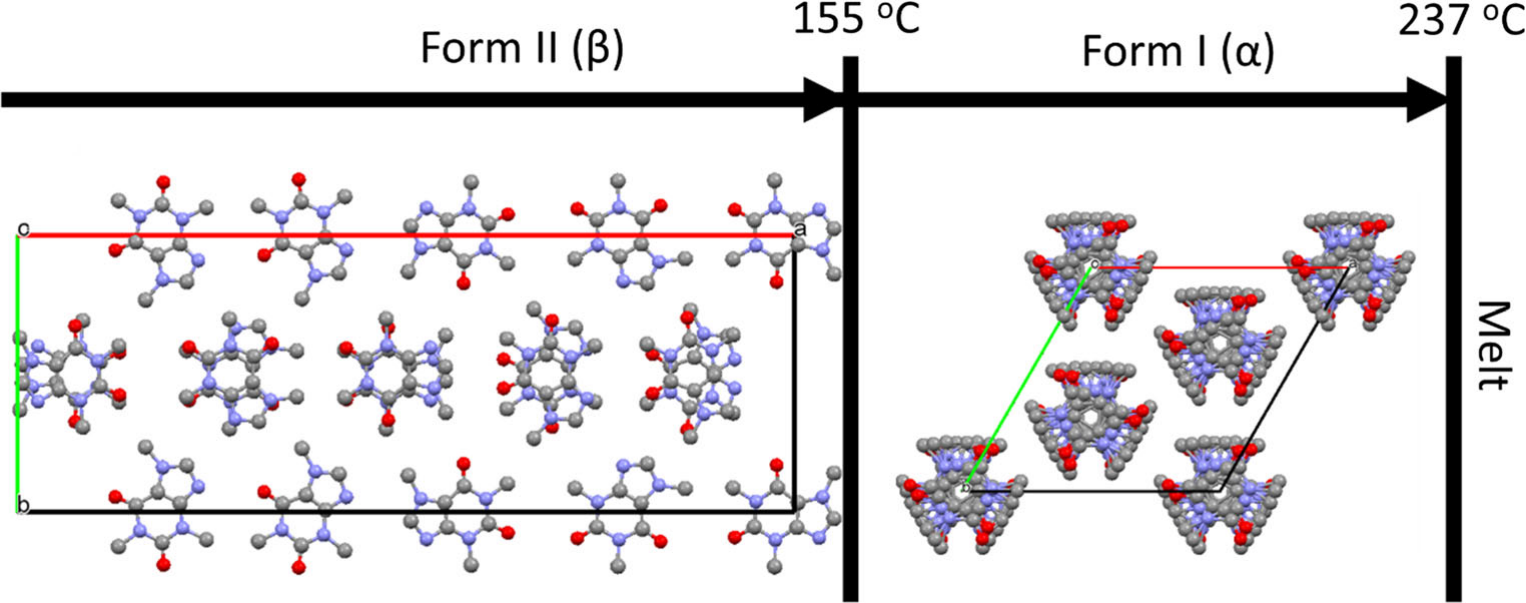
Crystal structures of anhydrous caffeine polymorphs, along the crystallographic *c*-axis. Temperatures and nomenclature are taken according to the literature [40]. The α-form is highly disordered about each crystallographic site

In this study, we make use of a pharmaceutically active compound, caffeine. Caffeine has been the subject of immense interest to the solid-state research community [28, 39–42] and has served as a fundamental example of the benefits of co-crystallisation on the physico-chemical properties of drug materials [43]. This compound exists in a variety of solid forms, for which the structures of two anhydrous polymorphs and one monohydrate are known, Fig. 1 [44, 45]. Both anhydrous polymorphs contain highly disordered caffeine molecules. It has been previously demonstrated that milling of both anhydrous forms leads to a metastable phase [39]. While the structure of this metastable phase remains unknown, the XRPD patterns suggest that the same phase is formed on milling either of the two anhydrous polymorphs. The present study examines the effect of milling parameters on the transformation of the stable form, caffeine II (CAFII), to the metastable intermediate state. In doing so, we present the first example of in situ real-time X-ray powder diffraction applied to a single-phase polymorphic transformation.

## Experimental

### Materials

Anhydrous caffeine (Sigma-Aldrich, 99%) was used as supplied, without further purification. Polymorph purity was confirmed by X-ray powder diffraction.

### Mechanical treatment

All milling experiments were performed with a MM400 Retsch ball mill, modified for purpose on a synchrotron beam line. For each experiment, 300 mg of pure anhydrous caffeine powder was used. Samples were loaded into Perspex milling vessels (internal volume 14.5 mL), which have been previously demonstrated to be suitable for in situ X-ray diffraction experiments [15, 16, 19, 46]. Jars were generated in line with the engineering specifications in [47]. A stainless steel ball of 1.4 g (7 mm diameter; small), 3.4 g (10 mm diameter; medium) or 13.4 g (15 mm diameter; large) was added to the vessel, depending on the experiment. Details are provided in the main text. The need for use of only a single ball in studying mechanochemical kinetics is necessary to eliminate stochastic ball–ball collisions [48]. Unless otherwise stated, milling was performed at 30 Hz. All experiments were done without deliberate addition of liquids, though without special drying of the surrounding air.

### Diffraction

All in situ diffraction experiments were performed on the ID-11 beam line at the European Synchrotron Radiation Facility (ESRF). Monochromatic X-rays of wavelength 0.141696 Å were used. Powder patterns were collected every 0.4 s. Data were averaged by summing 10 detector frames, giving a total time resolution of 4 s.

### Data processing

The 2D data were integrated using the PyFAI azimuthal integration method [49, 50]. Data were subsequently background corrected using the Sonneveld [51] scheme available in the Powder3D software [52]. Patterns were subsequently normalised to a total intensity of 1 to eliminate effects of varying powder in the X-ray [19]. This resulted in no change in the relative intensity of peaks within each pattern. The second derivative curves were plotted.

## Results and discussion

As the structure of the metastable intermediate phase remains unknown, it was not possible to conduct Rietveld refinement of the data obtained from in situ X-ray diffraction patterns. For the anhydrous phase of CAFII, the major feature in the diffraction pattern is a pair of Bragg peaks at *d*-spacing = 3.30 and 3.38 Å, ESI. Instead, in the intermediate phase these merge into a single, well-defined peak [39]. It is therefore possible to use this transformation to monitor the approximate rate of the transformation. Analysis of the background-corrected XRPD profiles (ESI) does not unambiguously allow detection of the second Bragg peak of the reactant phase. This is particularly true for partially converted samples, where the two reactant peaks are no longer well resolved. Instead, we make use of the derivative properties of curves. That being that the curvature of a peak is proportional to its height and that small ‘humps’ (e.g. shoulders) can be numerically detected. In this method, we are able to identify any traces of the second reactant peak, including the existence of small shoulder peaks. Provided minimal broadening of the product peak occurs; one therefore expects a complete conversion to result in a plateau at 0. For all points at which the curve sits below zero, a reactant peak remains.

We do note that this method cannot offer a direct comparison between different samples and only suggests a relative, internal quantification. That said, as the curvature directly reflects the shape of the Bragg peak and thus the crystallinity of the material, powder beginning from the same stock sample should have approximately the same initial value. We also note that small fluctuations can be expected due to statistical broadening from powder position within the vessel.

### Mechanical treatment of caffeine form II—30 Hz

**Figure 2:**
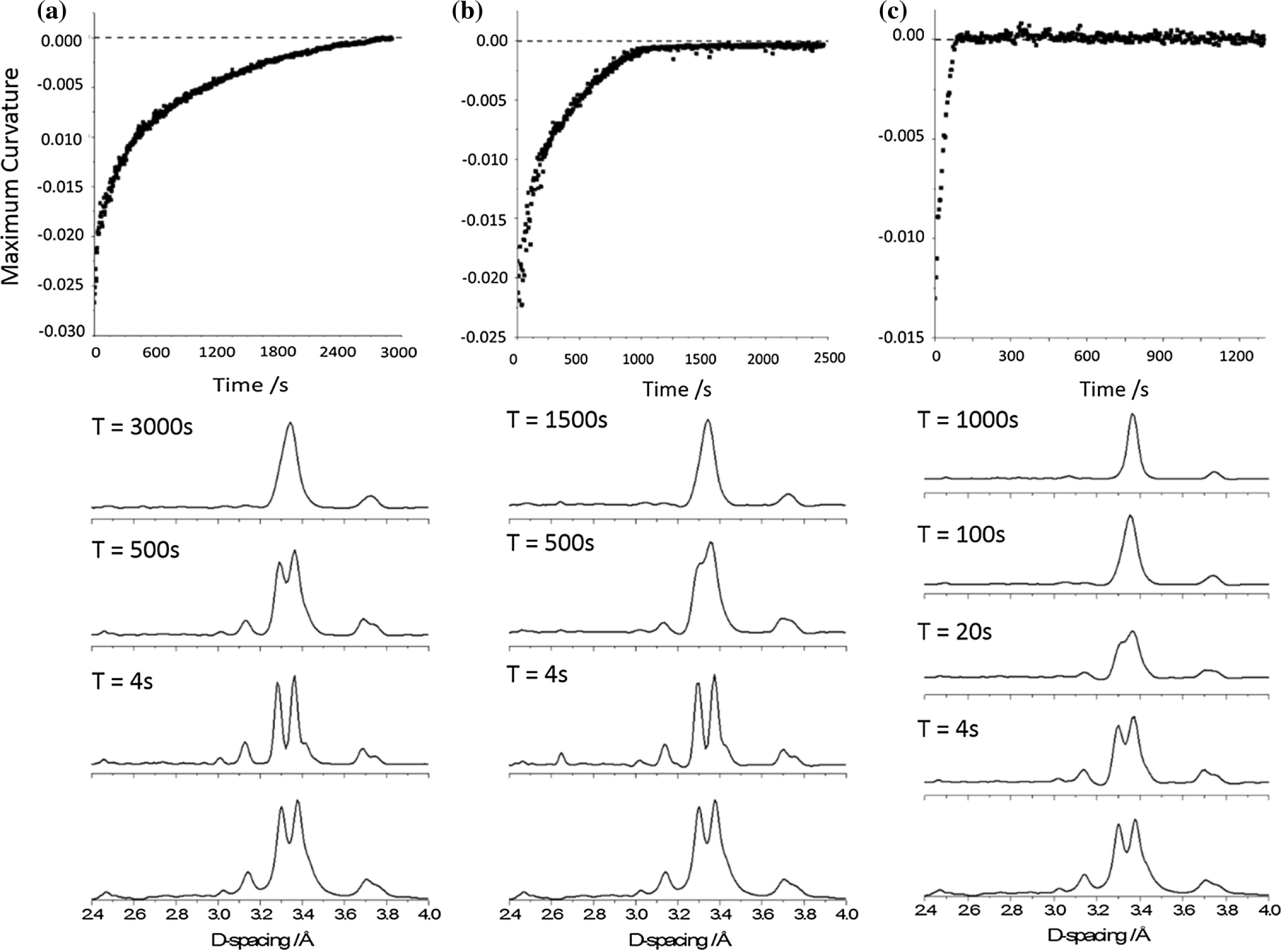
Transformation of CAFII by ball milling at 30 Hz with **a** 7 mm ball, **b** 10 mm ball and **c** 15 mm ball. The conversion profile is given (top), along with excerpt XRPD patterns at the identified time steps. The bottom XRPD profile is simulated CAFII in each case

Samples of CAFII were subjected to ball milling under three experimental conditions. In the first, the sample was milled at 30 Hz with a single small ball (7 mm diameter, 1.4 g), Fig. 2a. The reaction profile follows the expected general structure for a finite system and is consistent with observation of the raw XRPD profiles. The early stages of the transformation are marked by rapid conversion to the metastable intermediate phase. There is a high initial rate of conversion for the first ca 350 s, while the proportion of reactant remains high and each powder-ball impact can be assumed to be capable of a reactive collision. The profile is then seen to slow considerably as the reactant is consumed and many ball–particle collisions instead involve the product phase. A plateau is finally found to appear following ca 45 min of continued milling, Table 1. In contrast to many multi-phase kinetic profiles, we note that the polymorphic conversion does not exhibit an induction period. That is that nucleation of the product phase is not the limiting step. This further suggests that initiation of the polymorphic conversion is not dependent on a build-up of energy or temperature, but instead occurs as a direct result of ball–particle collisions.

**Table 1:** Characteristics of milling balls and the resulting times to plateau

Ball mass (g)	Ball diameter (mm)	Ball surface area (mm^2^)	Approx. plateau time (s)
1.4	7	153.94	2850
3.4	10	314.16	1000
13.4	15	706.86	100

Mathematically, any rate that is dependent upon the quantity of a reactant component can be described by a series of exponential equations. Relatively simple exponential functions have been previously derived for multi-component mechanochemical reactions, proving to describe a range of mechanochemical systems [48, 53, 54]. Typically, if only a single process is dominant, a single function can be employed of the form:

1 $$ \frac{{{\text{d}}\alpha }}{{{\text{d}}t}} \propto f\left( \alpha \right)\exp \left( \tau \right) $$

where the conversion quantity, α, is dependent on an appropriate kinetic law, *f(α)*, and a ratio of required and available energies, *τ*. In terms of mechanochemistry, provided sufficient energy is available from an impact, and τ can be approximated as the time-dependent availability of the perturbing energy (i.e. pulses associated with ball–particle impacts). This differs from solution chemistry where the energy profile of the activating energy remains constant. In this view, the conversion rate is proportional to the rate of the chemical or physical transformation itself, as well as the time-dependent injection of energy that is required to instigate the transformation.

**Figure 3:**
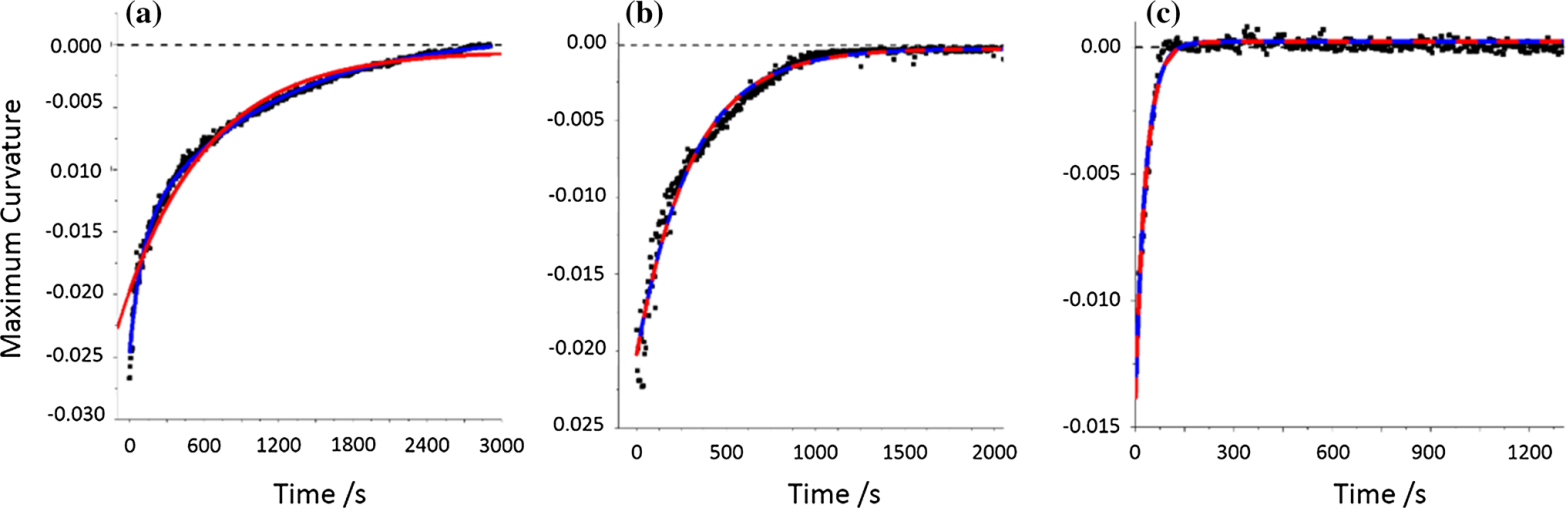
Fitting of multi-phase reaction equations to rate profiles of milling at 30 Hz with **a** small ball, **b** medium ball and **c** large ball. In each case, a single exponential fit red and double exponential fit blue are given

For the milling of CAFII, the rate profile of the transformation cannot be modelled using a simple one-component exponential growth function. A single time constant thus cannot be used to model this transformation. Instead, it is found that a two-phase equation perfectly reproduces the rate profile, Fig. 3a. This equation takes the form,

2 $$ \frac{{{\text{d}}\alpha }}{{{\text{d}}t}} \propto f'\left( \alpha \right)\left( {1 - \exp \left( {\tau^{\prime}} \right)} \right) + f''\left( \alpha \right)\left( {1 - \exp \left( {\tau ''} \right)} \right) $$

with each exponential function taking nearly equal weighting in the fit (1:0.6).

The need to employ such an equation highlights the qualitatively observed *fast* and *slow* regimes. When milling with the small milling ball, the former regime is over tenfold more rapid than the slow regime. Little physical interpretation can be made from the use of such mathematic constructs in the absence of deeper investigation of an underlying physical model.

However, given current understanding of mechanochemical processes, a plausible mechanism can be discussed. By studying a single-phase system, the need to form reactive contacts is eliminated. Noting that a mechanochemical reaction can occur only on contact with the milling ball, it follows that mass transport remains crucial in the originally single-phase systems, which become multiple-phase as the product phase appears. As the quantity of reactant decreases, the probability that it sits in the direct path of the milling ball at each impact is reduced. This leads to a notable decrease in the conversion rate, limited by mixing.

An increase in the ball mass by approximately twofold (10 mm diameter, 3.4 g) leads to a drastic shift in the rate of polymorphic transition, Fig. 2b. Again, the same three profile stages are observed. There is a rapid initial conversion, followed by a slowing of the transformation, and finally a plateau is reached. In this case, the plateau is found to arise by approximately 16–17 min of continued milling, Table 1. This is an approximately threefold increase in the rate of conversion as compared with the previous case. We can again fit the reaction profile to the same multistage equation, Eq. (2**)**, Fig. 3b. In contrast to milling with the smaller ball, the rate constants associated with the ‘fast’ and ‘slow’ regimes in this case differ only fivefold. However, the weightings of each exponential become 4:1. In fact, the profile can be fit by employing only the first exponential function, Fig. 3b. This suggests that the kinetic restriction imposed by mass transport is considerably less with the medium sized ball.

Finally, increasing the ball mass further (15 mm diameter, 13.4 g) yields a notably larger change than before, Fig. 2c. It is first noted that the initial powder pattern (*T* = 4 s) already exhibits notable merging of the reactant peaks at d-spacing of 3.30 and 3.38 Å, Fig. 3c. This suggests that after only four seconds of milling, notable conversion has already taken place. The conversion profile observed for the transformation with the largest ball appears to have only two stages: a very rapid transformation, followed by the plateau. There is no notable slowing of the conversion rate in this case. The conversion is notably quicker than the previous cases, with the plateau reached in only ca 100 s, Table 1. This is an approximately tenfold increase in conversion rate, with only fourfold increase in ball mass. Consistent with the previous two examples, we again fit the reaction profile to a multi-phase reaction, Eq. (2). Here, both exponential components adopt exactly the same form and weighting. Thus, the equation reduces exactly to a single function reaction equation. This suggests that there is no limitation imposed by mixing, and Eq. (2) reduces back to a single-component reaction model.

It is clear that the ball characteristics play an important role in driving the rate of this single-phase transformation, and their influence on the transformations is similar to its effect on multi-component reactions. However, the mechanism of this dependence is not clear. In the previous literature, it is generally suggested that the important factor of the milling ball is its mass [17] and that the rate of conversion is proportional to the energy imparted by the associated impact. This may be true for some reactions [55, 56]. In the present case, ball bass may be associated with more rapid comminution and an increase in the generation of defects per impact. These defect sites and surfaces are known to permit nucleation of polymorphic transitions. However, the lack of induction period suggests that any such nucleation must occur more rapidly than the timescales studied here. An additional factor that one should consider is the area of impact that is associated with each milling ball. That is to note that the number of impacted particles will greatly increase upon increasing the size of the milling ball. This, in turn, reduces any limiting effects of mixing, even in the case of an originally single-phase system. Interestingly, the previous works on the kinetics of mechanochemical reactions have identified a rate-limiting step to be generation of new reactive interfaces, according to an exponential function [54]. When ball–particle interfaces are considered, the rate of contact formation depends on *both* ball surface area and the rate of comminution of the powder sample. The exact role of the milling ball is certainly complex, with the reaction rate depending partially on the energy imposed by each milling ball, the area of impact, the depth of mechanical disturbance on a particle and even the rate of particle size reduction. With current technologies, it is not yet possible to identify which factors are dominant.

### Mechanical treatment of caffeine form II—25 Hz

**Figure 4:**
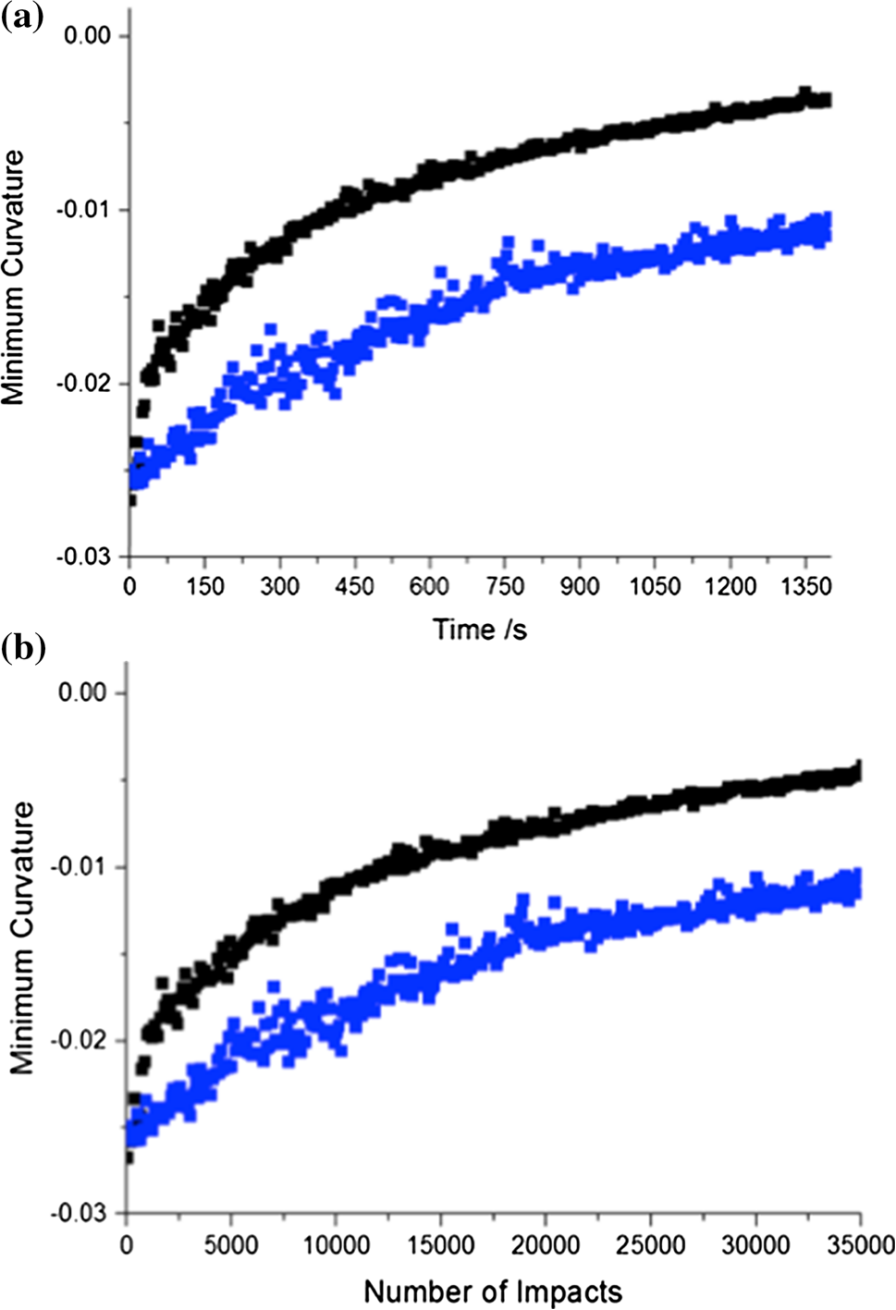
Comparison of conversion rates by milling at 25 Hz (blue) and 30 Hz (black). Milling is performed with the smallest milling ball (1.4 g). **a** The rate of transformation of CAFII as a function of time and **b** normalised for the theoretical number of impacts at each milling frequency

As a comparison, a small milling ball was also used to mill CAFII at 25 Hz. As observed at 30 Hz, there is no visible induction period. The transformation begins immediately, albeit with a considerably slower rate than at 30 Hz, Fig. 4a. In the first instance, this is unsurprising as the number of reactive impacts must be correspondingly less when milled at 25 Hz. However, normalisation by the ideal number of impacts continues to display a difference in the rate of conversion, Fig. 4b. Noting the stochastic motion of a milling ball within a vessel, it is impossible to know the exact number of impacts during any given experiment. That said, it is remarkable that the curves for milling at 25 Hz and 30 Hz run parallel except for the initial stages of the transformation. This is particularly apparent when the normalisation by the number of impacts is accounted for. The reason for early divergence of rates is likely due to the energy of the impacts associated with each milling frequency, which directly affects the rate of particle comminution and thus mixing.

## Conclusions

In contrast to solution and gas-phase processes, a mechanochemical reaction occurs only at particle–particle–milling ball interfaces. As such, the rate-determining step in multi-component mechanochemical reactions is formation of reactive interfaces. This hindrance can be removed by studying mechanochemical conversion of single-phase systems. Following the rate of polymorphic transformation of CAFII under milling revealed a sizable dependence between milling ball size and the rate of the transformation. Surprisingly, the rate of transformation cannot be modelled using a time-independent rate equation. This suggests that the dominating rate-determining step changes with time. It can be suggested that in the early stages of the reaction, all collisions occur between the milling ball and reactant CAFII. As the reaction proceeds, however, the probability of reactant-ball collisions decreases, and the rate becomes dominated by mass transport (mixing), since the system becomes two-phase as soon as the product is formed. The time dependence of this evolution depends on the milling ball size. It is not yet possible to unambiguously determine the root of this effect. From an energy perspective, larger and heavier milling balls lead to more rapid comminution and increased generation of defects, sites at which polymorphic transitions can nucleate. Larger milling balls also have larger surface areas and as such are less affected by poor mixing. The true effect is likely the result of a combination of these two complex phenomena. Model experiments with balls of variable size and constant mass, or alternatively, variable mass and constant size, will shed more light on the problem. Single-phase systems allow drastic reduction in the complexity of the mechanochemical problem under investigation. By employing such systems, new insight can be obtained with regard to the fundamental parameters of mechanochemical processes. This understanding is crucial for the selective and targeted design of mechanochemical reactions.

## Electronic supplementary material

Below is the link to the electronic supplementary material.

Supplementary material 1 (DOCX 445 kb)
