# Supplementary material for: The effect of ball mass on the mechanochemical transformation of a single-component organic system: anhydrous caffeine
Source: J Mater Sci. 2018 Apr 20;53(19):13380–9. doi: 10.1007/s10853-018-2324-2 (PMC6434987; doi:10.1007/s10853-018-2324-2)
Supplement: Supplementary file 1 — Supplementary material 1 (DOCX 445 kb) [file 10853_2018_2324_MOESM1_ESM.docx]

Electronic Supplementary Information:

The Effect of Ball Mass on the Mechanochemical Transformation of a Single Component Organic System: Anhydrous Caffeine

Adam A.L. Michalchuk,*^a,b,c^ Ivan. A. Tumanov,^a,d^ and Elena. V. Boldyreva*^a,d^

1. **Simulation of XRPD profile for Form II Caffeine**

The simulated X-ray powder diffraction pattern for CAFF-II indicates two unique peaks at d-spacing of 3.30 Å and 3.378 Å, Figure S1.

Figure S1: Simulated XRPD pattern for CAFF-II.

1. ***In situ* Real-Time X-ray Powder Diffraction of Ball Milling Anhydrous Caffeine**

The evolution of the phase transition in CAFII can be followed by the merging of the two Bragg peaks at d-spacings of *ca* 3.30 and 3.38 Å, Figure S2. It is clearly seen that this merging (and thus transformation) occurs slowly for the small milling ball, Figure S2A, increasing with ball size. The transformation occurs very rapidly with the large milling ball, Figure S2C.


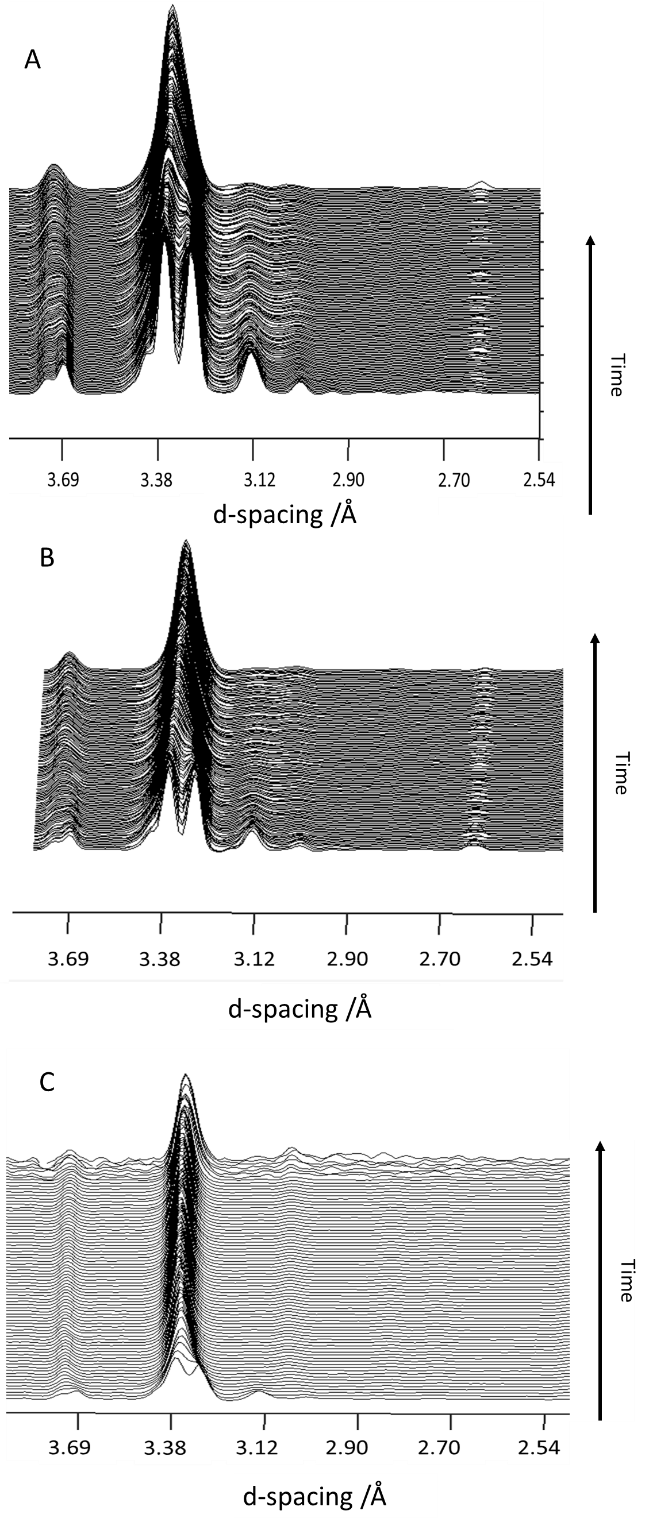


Fig S2: Time resolved XRPD profiles for ball milling of anhydrous CAFF-II. Milling was performed at 30 Hz using a small (A), medium (B), and large (C) milling ball. Dimensions given in the main text.

1. **Exponential Functions for Profile Fitting**

Curve fitting of the exponential equation

$y=Aexp\left( 1-x/t1 \right)+Bexp\left( 1-x/t2 \right)$ Equation 1

can be used as a general 2-regime reaction equation. For the present cases the fit parameters given in Fig 3 of the main text are given in Table S1.

Table S1: Fitting parameters for Equation 1, used to model conversion profiles for milling of anhydrous caffeine.

|  | A | T1 | B | T2 |
| --- | --- | --- | --- | --- |
| Small ball | 0.00958±1.61E-4 | 99,241±3.34 | 0.01602±9.26E-5 | 1092.281±19.58 |
| Medium ball | 0.00437±5.14E-4 | 69.299±11.72 | 0.01707±5.29E-4 | 358.492±9.62 |
| Large ball | 0.00702±1E-6 | 32.955±3.11 | 0.00702±1E-6 | 32.955±3.11 |

The fit values of the one-component equation,

$y=Cexp\left( x/t3 \right)$ Equation 2

for each curve are given in Table S2.

Table S2: Constants associated with Equation 2 for each milling ball used.

|  | C | T3 |
| --- | --- | --- |
| Small ball | -- | -- |
| Medium ball | -0.01984±.29E-4 | -309.459±3.48 |
| Large ball | -0.01404±3.53E-4 | -32.956±1.27 |

It is seen that Equation 1 for the large ball reduces exactly to the single exponential function, while fitting for the medium ball requires an intermediate rate constant. The fit for the small ball cannot be reduced to a single exponential function.
